# Supplementary material for: Vagal nerve stimulation triggers widespread responses and alters large-scale functional connectivity in the rat brain
Source: PLoS One. 2017 Dec 14;12(12):e0189518. doi: 10.1371/journal.pone.0189518 (PMC5730194; doi:10.1371/journal.pone.0189518)
Supplement: S1 File — This file (zip format) contains the ICA-derived network responses to VNS. (ZIP) [file pone.0189518.s001.zip › shared data/readme.rtf]

ICA maps, time series, and brain images were included here.	sharedData_brain.nii.gz  		- brain anatomical images	sharedData_ICA_map.nii.gz 	- threshold ICA maps which significantly respond to VNS	sharedData_ICA_timeSeries.mat		ts	- averaged time series for each ICA component 		stim	- timing of stimulation block 
